# Targeting the KIFC1‐SRSF3‐PKM Axis Suppresses Cervical Cancer Glycolysis and Enhances Radiosensitivity

**DOI:** 10.1111/jcmm.71373

**Published:** 2026-09-20

**Authors:** Junxia Liu, Jiahao Li, Haiting Zhou, Wentao Ha, Xinyu Wu, Xiangdong Liu, Yizhi Jiang, Chen Gong, Yi Cheng, Qingxu Liu, Tengfei Chao, Huihua Xiong

**Affiliations:** ^1^ Department of Oncology Tongji Hospital, Tongji Medical College, Huazhong University of Science and Technology Wuhan Hubei China; ^2^ Department of Oncology, the First Affiliated Hospital of Nanchang University Nanchang University Nanchang Jiangxi China

**Keywords:** alternative splicing, cervical cancer, glycolysis, KIFC1, radiosensitivity

## Abstract

Metabolic reprogramming towards enhanced glycolysis contributes to cervical cancer progression and radioresistance, but its upstream regulatory mechanisms remain poorly understood. Here, we investigated the role and underlying mechanisms of kinesin family member C1 (KIFC1) in glycolytic reprogramming and radiosensitivity in cervical cancer. KIFC1 expression and clinical relevance were analysed using public datasets and cervical cancer tissue microarrays. In vitro and in vivo assays were performed to evaluate the effects of KIFC1 on tumour growth, glycolysis and radiosensitivity. Transcriptomic and molecular interaction analyses were performed to define the KIFC1‐SRSF3‐PKM regulatory axis. KIFC1 was upregulated in cervical cancer tissues and associated with poor clinical outcomes. KIFC1 depletion inhibited tumour growth, reduced glycolytic activity and enhanced radiosensitivity both in vitro and in vivo. Mechanistically, KIFC1 may facilitate the nuclear localization of the splicing factor SRSF3, thereby influencing PKM alternative splicing. Loss of KIFC1 resulted in cytoplasmic retention of SRSF3 and a switch from the pro‐glycolytic PKM2 isoform to PKM1, leading to impaired glycolysis. This splicing‐mediated metabolic reprogramming was a key determinant of radiosensitization and could be partially reversed by pharmacological activation of PKM2. These findings identify the KIFC1‐SRSF3‐PKM axis as a novel splicing–metabolic checkpoint governing radiosensitivity in cervical cancer and highlight KIFC1 as a promising therapeutic target for improving radiotherapy efficacy in cervical cancer.

**Abbreviations**
ATCC: American Type Culture Collection
BCA: bicinchoninic acid
CCK‐8: cell counting kit‐8
Co‐IP/MS: co‐immunoprecipitation and mass spectrometry
DEGs: differentially expressed genes
DFS: disease‐free survival
GO: gene ontology
IHC: immunohistochemistry
KEGG: Kyoto Encyclopedia of Genes and Genomes
KIFC1: kinesin family member C1
OS: overall survival
PKM: pyruvate kinase M
RIP: RNA extraction and real‐time PCR
RNA‐seq: RNA sequencing
RT: room temperature
RT– qPCR: RNA extraction and quantitative real‐time PCR
siRNAs: small interfering RNAs
SRSF3: serine/arginine‐rich splicing factor 3

## 1 Introduction

Cervical cancer remains a leading cause of cancer‐related mortality, with radiotherapy as a cornerstone of treatment [1, 2, 3]. However, the frequent development of radioresistance limits therapeutic efficacy [4, 5, 6, 7]. Metabolic reprogramming towards aerobic glycolysis has emerged as a key facilitator of this resistance, enabling tumour cells to sustain proliferation, fuel DNA repair and mitigate radiation‐induced oxidative stress [8, 9, 10, 11]. Despite its significance, the molecular regulators that orchestrate glycolysis‐dependent radioresistance in cervical cancer remain poorly defined. Here, we identify kinesin family member C1 (KIFC1) as a critical regulator of a splicing–metabolic checkpoint that links intracellular transport, alternative splicing and glycolytic adaptation to radiosensitivity.

Pyruvate kinase M (PKM) is a central glycolytic enzyme subject to alternative splicing, producing PKM1 and PKM2 isoforms [12]. PKM2 is commonly associated with aerobic glycolysis and tumour progression, whereas PKM1 is more closely linked to oxidative metabolism [13, 14, 15, 16]. SRSF3, a prototypical serine/arginine‐rich splicing factor, promotes PKM exon 10 inclusion, shifting the isoform balance towards PKM2 and facilitating the Warburg effect. SRSF3 is a nucleocytoplasmic shuttling splicing factor whose activity depends on its nuclear localization and plays an important role in PKM alternative splicing [17]. However, the upstream mechanisms that regulate SRSF3 localization in cancer cells remain largely unexplored.

KIFC1 is a motor protein implicated in the directed transport of diverse intracellular cargoes [18, 19, 20, 21, 22]. It is overexpressed in multiple malignancies and implicated in tumour progression [23, 24, 25]. Given its transport‐related functions, we hypothesized that KIFC1 may mediate the nuclear import of SRSF3, thereby modulating PKM alternative splicing, glycolytic reprogramming and the radiation response in cervical cancer.

In this study, we investigated the role of KIFC1 in cervical cancer progression, glycolytic reprogramming and the radiation response. In particular, we examined whether KIFC1 modulates PKM alternative splicing via SRSF3 and evaluated the potential contribution of this pathway to radioresistance.

## 2 Materials and Methods

### 2.1 Bioinformatic Analysis

Publicly available transcriptomic datasets of cervical cancer were obtained from the Gene Expression Omnibus (GEO) database using the GEOquery package in R. Six expression resources were included in the analysis: GSE145976, GSE192804, GSE113942, GSE52903, GSE44001 and GSE67522. GSE145976 is a previously integrated cervical cancer transcriptomic dataset comprising 313 samples derived from five independent GEO cohorts (GSE9750, GSE7803, GSE6791, GSE63514 and GSE29570). Only samples annotated as cervical cancer or normal/control were retained for the present analysis. GSE192804 contains 43 RNA‐sequencing samples obtained from 14 patients, including primary tumour tissue (TT), paracarcinoma tissue (TP) and normal cervical tissue (NC); only TT and NC samples were retained, whereas TP samples were excluded. GSE113942 contains 14 samples, including seven cervical cancer and seven normal cervical tissues. GSE52903 contains 72 samples, including 55 cervical cancer and 17 non‐tumour cervical controls. GSE67522 contains 42 samples, including 20 cervical cancers, 11 HPV‐negative histologically normal cervical controls and 11 HPV16‐positive non‐malignant cervical samples; only cervical cancer and HPV‐negative normal samples were included in the tumour‐versus‐normal differential expression analysis. GSE44001 contains 300 cervical cancer samples without a corresponding normal control group and was therefore not independently subjected to tumour‐versus‐normal differential expression analysis.

For each dataset, the processed expression matrix and corresponding phenotype information were retrieved from GEO. Probe or transcript identifiers were mapped to official gene symbols using the corresponding platform annotations or the org.Hs.eg.db annotation package. Features without valid gene‐symbol annotations were removed, and when multiple probes or identifiers mapped to the same gene, a single representative feature was retained. For count‐based RNA‐sequencing datasets, expression values were transformed as log_2_(*x* + 1). For microarray datasets, the normalized expression values provided by GEO were used after probe‐to‐gene annotation.

Because the included cohorts were generated using different experimental platforms and study designs, differential expression analysis was performed independently within each dataset rather than by fitting a single model to the pooled expression matrix. This strategy prevented inter‐study platform effects from being directly confounded with the tumour‐versus‐control comparison. Within each dataset containing both cancer and control samples, differential expression between cervical cancer and control tissues was assessed using the limma package (v3.62.2) in R (v4.4.0). *p* values were adjusted for multiple testing using the Benjamini–Hochberg false discovery rate procedure, and genes with an adjusted *p*‐value < 0.05 were considered differentially expressed.

Kinesin family genes were subsequently extracted from the differential expression results. Their log_2_ fold‐change estimates from the individual cohorts were compiled into a cross‐dataset matrix to evaluate the direction and consistency of differential expression. Kinesin genes were prioritized according to their mean log_2_ fold change across evaluable datasets. Heatmaps were generated using ComplexHeatmap (v2.18.0), and scatter plots were generated using ggplot2 (v3.5.1).

### 2.2 Tissue Microarrays

We obtained cervical cancer tissue microarrays (1.5‐mm diameter) from Outdo Biotech. The tissues were formalin‐fixed and contained comprehensive clinical data, including gender, age, tumour stage and histology. Follow‐up information was available for 115 patients, all of whom were included in the correlation and survival analyses, including 36 deaths and 79 survivors. We conducted three preliminary experiments to determine optimal antibody dilutions. Immunohistochemical (IHC) staining was performed using KIFC1 antibody (1:200, #R24806; ZENBIO). IHC staining for KIFC1 was evaluated by two pathologists independently, blinded to clinical data. Staining was semi‐quantitatively scored based on intensity and area. Intensity was scored as 0 (negative), 1 (weak, light yellow), 2 (moderate, brown) or 3 (strong, dark brown). The area of positive staining was scored as 1 (0%–25%), 2 (26%–50%), 3 (51%–75%) or 4 (76%–100%). The final IHC score was calculated by multiplying intensity and area, yielding a total score ranging from 0 to 12. Patients were classified as having high or low KIFC1 expression based on the median score. Intergroup differences in overall survival (OS) and disease‐free survival (DFS) were analysed. All procedures involving human samples were conducted in accordance with the Declaration of Helsinki. All human tissue microarray samples used in this study were obtained from Shanghai Outdo Biotech Co. Ltd., for which written informed consent was obtained from all donors at the time of tissue collection by the original providers. The use of these de‐identified tissue samples was approved by the Ethics Committee of Shanghai Outdo Biotech (approval number: SHYJS‐CP‐1710003).

### 2.3 Cell Culture

HeLa (RRID: CVCL_0030) and SiHa (RRID: CVCL_0032) cell lines were sourced from the American Type Culture Collection (ATCC), authenticated, maintained under ATCC‐recommended conditions and tested for mycoplasma upon thawing. The cells were maintained in DMEM with high glucose medium (#G4511; Servicebio) containing 10% foetal bovine serum (#6123139; Gibco). The cells were seeded in cell culture dishes under sterile conditions at 37°C with 5% CO_2_. Subsequent experiments were performed when cells reached 80%–90% confluence.

### 2.4 Cell Transfection

KIFC1‐overexpressing and KIFC1‐silencing lentiviruses, along with a control lentivirus, were produced by Genechem. Lentiviral shRNAs targeting KIFC1 (sh‐KIFC1#1: GTCCAGCCTGGGACTTAAACT; sh‐KIFC1#2: TGTCACCAATGCTCGATATCT) were used to knock down KIFC1. For KIFC1 overexpression, a CRISPR activation (CRISPRa) system employing lentiSAMv2/lentiMPHv2 vectors was used, with an sgRNA targeting the KIFC1 promoter (sequence: GCAGAGCGCGGTGAGAGTGGG) to induce endogenous KIFC1 expression. Cells (5 × 10^5^) were transduced in the presence of 5 μg/mL polybrene (#40804ES; Yeasen) in six‐well plates and selected with 3 μg/mL puromycin (#A610593; Sangon Biotech) for 72 h. Stable KIFC1‐overexpression and KIFC1‐knockdown cells were confirmed by RT‐qPCR and Western blot.

Human SRSF3‐targeting small interfering RNAs (siRNAs) were generated by Sangon Biotech. The siRNAs used were si‐SRSF3#1 (antisense sequence: UACAUAAACCUUACAGUCCTT) and si‐SRSF3#2 (antisense sequence: UAGUGUUCUUCCAUCUAGCTT), which were transfected together with plasmids using BOLG‐RNATM transfection reagent (#BOLG101; Biology) according to the manufacturer's protocol, followed by incubation for 24–48 h. The siRNA‐mediated transient silenced cells were validated by RT‐qPCR and Western blot. For SRSF3 overexpression, cells were transfected with an SRSF3 overexpression plasmid (OE SRSF3; Sangon Biotech) using Neofect DNA Transfection Reagent (#TF20121201; Neofect Biotech) according to the manufacturer's protocol. Cells were incubated for 24–48 h after transfection before subsequent experiments. SRSF3 overexpression was confirmed by RT‐qPCR and Western blot.

### 2.5 Reagents and Treatment

TEPP‐46 (#HY‐18657; MCE), a selective PKM2 activator, was used in this study. Cells were treated with TEPP‐46 at a concentration of 10 μM for 24 h prior to irradiation. The treatment regimen was selected based on previously published studies and its pharmacological profile, ensuring effective PKM2 activation while remaining below cytotoxic levels.

### 2.6 Cell Counting Kit‐8 (CCK‐8) and Colony Formation Assays

We assessed cell proliferation through the CCK‐8 assay. Cells were seeded at 2000 cells per well in quintuplicate and viability was assessed at 0, 24, 48, 72 and 96 h. After incubation with the CCK‐8 solution (NCM Biotech, China) for 2 h at 37°C under light‐protected conditions, OD450 was measured to generate growth curves. For the clonogenic assay, 1000 cells/well were seeded in six‐well plates and maintained under standard culture conditions (37°C, 5% CO_2_) for 14 days. Colonies were fixed with 4% paraformaldehyde for 40 min, stained with 0.1% crystal violet for 2 h, and finally counted and imaged to assess colony formation.

### 2.7 Wound Healing and Transwell Assays

For the wound healing assay, once cells reached approximately 90% confluence in culture plates, a linear scratch was generated with a pipette tip. Cell migration was documented by imaging at 0 and 24 h. For Transwell assays, 5 × 10^4^ cells were seeded into Transwell chambers (#354480; Corning) and cultured for 24 h. Migrated cells on the lower surface were fixed with 4% paraformaldehyde, stained with 0.1% crystal violet and counted for quantification.

### 2.8 RNA Sequencing (RNA‐Seq) and Bioinformatic Analysis

HeLa cells harbouring sh‐KIFC1 or sh‐NC were cultured in six‐well plates in triplicate and harvested for RNA extraction when cell confluence reached approximately 90%. Total RNA was isolated using TRIzol reagent (#G3013‐100ML; Servicebio) following the manufacturer's instructions, and RNA quality was assessed with a NanoDrop 2000 spectrophotometer. Sequencing libraries were constructed and subsequently sequenced on the Illumina NovaSeq 6000 platform. DEGs were defined using the thresholds of fold change ≥ 1.5 and FDR < 0.05. DEGs were functionally annotated using Gene Ontology (GO) and Kyoto Encyclopedia of Genes and Genomes (KEGG) enrichment analyses.

### 2.9 Glucose Consumption, Lactate Production and ATP Levels Assay

Glucose consumption, lactate production and ATP levels were quantified using the corresponding detection kits (Nanjing Jiancheng Bioengineering Institute, #A154‐1‐1, #A019‐2‐1, #A095‐1‐1). For glucose consumption, cells were seeded at equal densities and cultured in complete medium. After 24 h, culture medium was collected, and glucose concentration was measured using the glucose assay kit according to the manufacturer's instructions. Glucose consumption was calculated as the difference between the initial glucose concentration in fresh medium and the remaining glucose concentration after cell culture and normalized to the number of viable cells. Lactate production and ATP levels were measured in cell lysates according to the manufacturer's protocols, and data were similarly normalized to viable cell number to ensure comparability.

### 2.10 Co‐Immunoprecipitation and Mass Spectrometry (Co‐IP/MS)

HeLa and SiHa cells (1 × 10^7^) were harvested and disrupted in NP‐40 (#HY‐K1002; MCE) containing PMSF (#G2008‐1ML; Servicebio), followed by incubation on ice for 30 min. The lysates were centrifuged at 12,000 *g* for 25 min at 4°C. Supernatants were divided into input, IgG and IP groups. Input samples were boiled in protein loading buffer (#WB2001; NCM Biotech). For immunoprecipitation, protein lysates were mixed with anti‐KIFC1/SRSF3 or control IgG antibodies at 4°C overnight, then incubated with precleared protein A/G agarose beads (#HY‐K0202K; MCE) for 2 h at room temperature (RT). After washing and removal of beads, the immunoprecipitated proteins were boiled in loading buffer and subsequently detected via immunoblot or mass spectrometry.

### 2.11 Nuclear and Cytoplasmic Protein Extraction

We extracted nuclear and cytoplasmic fractions from cervical carcinoma cells using the Nuclear and Cytoplasmic Protein Extraction Kit (#EX1470; Solarbio), according to the manufacturer's protocol. Cells were suspended in ice‐cold buffer A, kept on ice for 20 min and centrifuged at 2000 *g* for 5 min at 4°C to isolate the cytosolic fraction. The resulting pellet was then resuspended in cold Extraction Buffer B, incubated and centrifuged at 12,000 *g* for 10 min at 4°C to collect the nuclear fraction. Protein concentrations were quantified with the bicinchoninic acid (BCA) assay (#AR0197; Boster), and samples were processed for Western blotting. Fraction purity was confirmed by GAPDH and histone H3 expression.

### 2.12 Cellular Immunofluorescence Assay

Cells were fixed with 4% PFA for 15 min at RT and permeabilized with 0.2% Triton X‐100 for 10 min. After blocking with 5% BSA for 1 h, cells were incubated with a primary antibody overnight at 4°C. Following three washes with cold PBS, cells were incubated with a fluorescently labelled secondary antibody (#GB21303; Servicebio) for 1 h at RT in the dark. Nuclei were counterstained with DAPI (#G1012‐10ML; Servicebio), and cells were embedded in an anti‐fade mounting medium for observation under a fluorescence microscope or a confocal laser‐scanning microscope. The primary antibodies used for immunofluorescence assay are listed as follows: SRSF3 (1:500, #ab198291; Abcam); γ‐H2AX (1:200, #R24564; ZENBIO); 53BP1 (1:100, #R381816; ZENBIO); and RAD51 (1:500, #R25533; ZENBIO).

### 2.13 RNA Extraction and Quantitative Real‐Time PCR (RT–qPCR)

RNA was extracted with TRIzol (#G3013‐100ML; Servicebio) in line with the manufacturer's protocol. First‐strand cDNA was synthesized using a reverse transcription kit (#R222‐01; Vazyme). Quantitative PCR was performed with ChamQ SYBR qPCR Master Mix (#Q411‐03; Vazyme) using cDNA templates, gene‐specific primers and nuclease‐free water. Relative gene expression levels were determined using the 2^−ΔΔCt^ method. All primer sequences were custom‐synthesized by Sangon Biotech (Table S1).

### 2.14 Western Blot

Proteins were extracted from cells or tissues in ice‐cold RIPA buffer (P0013B; Beyotime) containing protease inhibitor cocktail (#G2008‐1ML; Servicebio). Lysates were subjected to sonication and centrifugation at 12,000 rpm for 20 min at 4°C to remove debris. Protein concentrations were determined using a BCA assay (#AR0197; Boster), mixed with protein loading buffer (#WB2001; NCM Biotech) and boiled at 100°C for 5 min. The denatured proteins were resolved via SDS‐PAGE and blotted onto PVDF membranes (#IPVH00010; Millipore). Membranes were blocked in 5% BSA for 1 h at RT and probed with primary antibodies overnight at 4°C. Following washing, the membranes were incubated with HRP‐labelled Goat Anti‐Rabbit IgG (1:10,000, #GB23303; Servicebio) or Anti‐Mouse IgG (1:10,000, #GB23301; Servicebio) for 2 h at RT. Protein bands were detected by enhanced chemiluminescence and quantified using ImageJ software to evaluate relative protein expression levels. The primary antibodies employed for Western blotting are listed as follows: β‐actin (1:3000, #GB12001‐100; Servicebio); KIFC1 (1:1000, R24806; ZENBIO); SRSF3 (1:1000, #ab198291; Abcam); PKM1 (1:500, A18800; Abclonal); PKM2 (1:1000, A20991; Abclonal).

### 2.15 RNA Immunoprecipitation (RIP) Assay

RIP assays were carried out using a RIP kit (#Bes5101; Bersinbio) according to the manufacturer's instructions. Lysates from 3 × 10^7^ cells in RNase inhibitor‐supplemented buffer were collected, with a portion retained as input RNA. The lysates were divided into IP and IgG groups and exposed to 2 μL of anti‐SRSF3 antibody (#ab198291; Abcam) or control IgG overnight at 4°C. Immune complexes were captured with protein A/G‐conjugated magnetic beads and washed to eliminate non‐specific interactions. Bound RNA–protein complexes were treated with proteinase K to recover the associated RNAs, which were subsequently extracted and analysed by RT‐qPCR.

### 2.16 Flow Cytometric Analysis

Apoptotic cell death was determined in 1 × 10^6^ cervical cancer cells using an Annexin V‐FITC/PI Apoptosis Detection Kit (#40302ES20; Yeasen) according to the manufacturer's instructions. After labelling with Annexin V‐FITC and propidium iodide for 15 min at RT in the dark, cells were immediately analysed by flow cytometry. The FlowJo software was then applied to quantify apoptotic populations and distinguish early from late apoptotic cells.

### 2.17 IHC Analysis

Paraffin‐embedded tissues were sectioned using a microtome, deparaffinized and rehydrated through de‐paraffin liquid (#G1128; Servicebio) and graded ethanol series. Sections were then treated with the membrane‐breaking solution (#G1204; Servicebio) as recommended by the manufacturer. To quench endogenous peroxidase activity, tissue sections were incubated with 3% hydrogen peroxide for 10 min at room temperature.

Tissue sections were first treated with 5% BSA for 30 min to block non‐specific binding sites, followed by incubation with primary antibodies at 4°C overnight. After washing, HRP‐labelled secondary antibodies, including Goat Anti‐Rabbit IgG (1:10,000, #GB23303; Servicebio) or Goat Anti‐Mouse IgG (1:10,000, #GB23301; Servicebio), were applied for 1 h at RT to detect the bound primary antibodies. The immunoreactive signal was developed with 3,3′‐diaminobenzidine for 5 min, followed by brief nuclear counterstaining with haematoxylin for 30–60 s to provide contrast and facilitate microscopic observation. The slides were mounted and examined under a microscope. The primary antibodies applied were: KIFC1 (1:1000, #R24806; ZENBIO), Ki‐67 (1:100, #9027; Cell Signaling Technology), PKM1 (1:100, #A18800; Abclonal) and PKM2 (1:200, #A20991; Abclonal).

### 2.18 Subcutaneous Xenograft Model

BALB/c‐nu mice (female, 5 weeks old, 14–18 g) were supplied by GemPharmatech LLC (Jiangsu, China) and maintained under specific pathogen‐free conditions. Each animal was treated as an independent experimental unit. The cells were harvested and adjusted to a density of 5 × 10^7^/mL, after which 100 μL of the suspension was administered via subcutaneous injection into the right flank near the axillary area of each mouse. When the resulting tumours reached approximately 100 mm^3^ (volume = length × width^2^/2) on Day 7 post‐inoculation, 24 tumour‐bearing mice were randomly assigned to four experimental groups, including sh‐NC, sh‐KIFC1, sh‐NC + IR and sh‐KIFC1 + IR groups (*n* = 6 per group). Animals were stratified according to tumour volume and then allocated to treatment groups by computer‐generated randomization. Allocation concealment was maintained by an independent investigator who performed the assignment and disclosed group allocation only after enrolment was completed. Mice in the sh‐NC + IR and sh‐KIFC1 + IR groups underwent focal irradiation (6 Gy) at the tumour site. Tumour volumes were recorded every 3 days by an investigator blinded to group allocation. Animals were euthanised when humane endpoint criteria were met, defined as a tumour volume exceeding 1500 mm^3^ or sustained body weight loss > 20% for 48 h. Euthanasia was performed using intraperitoneal sodium pentobarbital (80 mg/kg), followed by cervical dislocation. Tumour tissues were collected. The study protocol was approved by the Institutional Animal Ethics Committee of Tongji Hospital, Tongji Medical College, Huazhong University of Science and Technology (approval number: TJH‐202411043). All experimental procedures followed the ARRIVE guidelines for reporting animal research.

### 2.19 Statistical Analysis

GraphPad Prism 8.0 was used to analyse all experimental data. Data showing a normal distribution, as assessed by the Shapiro–Wilk test (*p* > 0.05), are presented as mean ± standard deviation (SD). Differences between two groups were evaluated using a two‐tailed, unpaired Student's *t*‐test. Comparisons among multiple groups were performed using one‐way or two‐way ANOVA followed by appropriate post hoc tests. Experiments were independently repeated at least three times. A *p*‐value < 0.05 was considered statistically significant. Statistical significance is indicated as follows: *p < 0.05, **p < 0.01, ***p < 0.001, ****p < 0.0001.

## 3 Results

### 3.1 KIFC1 Is Upregulated in Cervical Cancer and Predicts Unfavourable Prognosis

**FIGURE 1:**
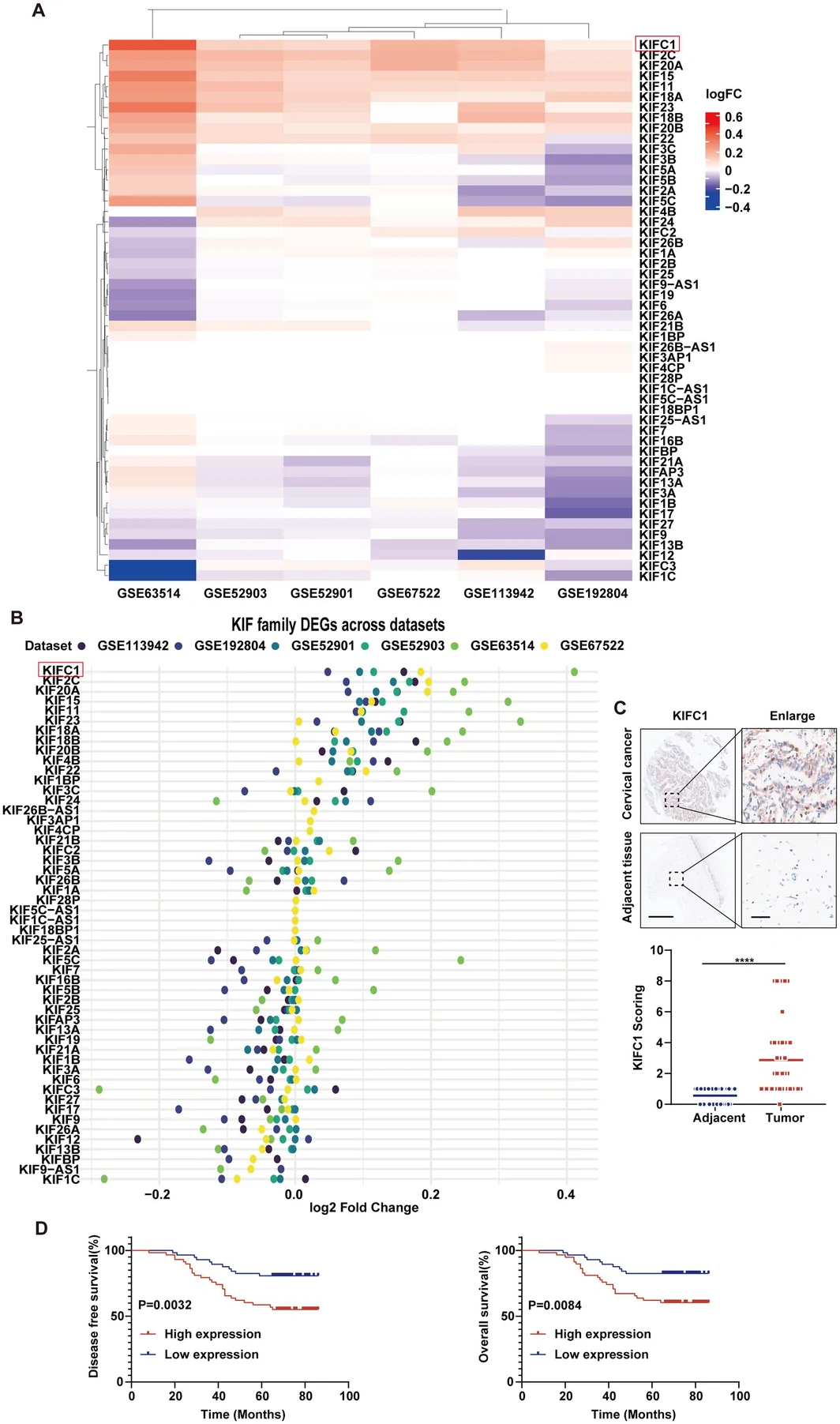
KIFC1 is upregulated in cervical cancer and associated with poor prognosis. (A, B) Heatmap and scatter plots showing KIFC1 expression in cervical cancer tissues across multiple GEO datasets. (C) KIFC1 levels in cervical cancer and paired adjacent non‐tumour tissues were evaluated by IHC analysis (scale bar: 50 μm). (D) Kaplan–Meier curves for DFS and OS in cervical cancer patients with high‐ and low‐KIFC1 expression (*n* = 115). Data are expressed as mean ± SD. Experiments were performed in triplicate. *p < 0.05, **p < 0.01, ***p < 0.001, ****p < 0.0001.

Kinesin motor proteins have emerged as key regulators of tumour initiation and malignant progression, as supported by accumulating evidence [23, 24, 25]. To identify kinesin family members potentially involved in cervical cancer, we analysed six GEO datasets (GSE113942, GSE192804, GSE52901, GSE52903, GSE63514 and GSE67522) and identified KIFC1 as one of the most significantly dysregulated kinesin family members in cervical cancer tissues compared with normal tissues (Figure 1A,B). Publicly available datasets were employed to analyse KIFC1 expression profiles, revealing its marked overexpression across more than 20 cancer types, including cervical cancer (Figure S1A). KIFC1 overexpression was further validated in cervical cancer tissues using GEPIA 2.0 analysis (Figure S1B). Additionally, UALCAN database analysis revealed that KIFC1 was markedly upregulated across cervical cancer subtypes and linked to lymph node metastasis (Figure S1C,D).

We next evaluated KIFC1 protein expression in a tissue microarray comprising 115 cervical carcinoma specimens and 39 adjacent non‐tumour tissues. Immunohistochemical analysis revealed that KIFC1 expression was significantly elevated in cervical carcinoma tissues compared with adjacent normal tissues (Figure 1C). Based on IHC evaluation, patients were stratified into high‐ and low‐KIFC1 expression subgroups (Figure S1E). Kaplan–Meier analysis showed that high KIFC1 expression was significantly correlated with poorer DFS and OS (Figure 1D). Our findings indicate that KIFC1 is overexpressed in cervical carcinoma and serves as a potential prognostic marker.

### 3.2 KIFC1 Knockdown Inhibits Cervical Cancer Cell Proliferation and Migration

To further investigate the function of KIFC1 in cervical carcinoma, we established stable KIFC1‐knockdown and KIFC1‐overexpressing HeLa and SiHa cell lines by lentiviral transduction. RT‐qPCR analysis indicated substantial downregulation of KIFC1 in knockdown cells and a marked increase in overexpressing cells, confirming efficient transfection. Western blot analysis further substantiated these results (Figure S2A–D).

**FIGURE 2:**
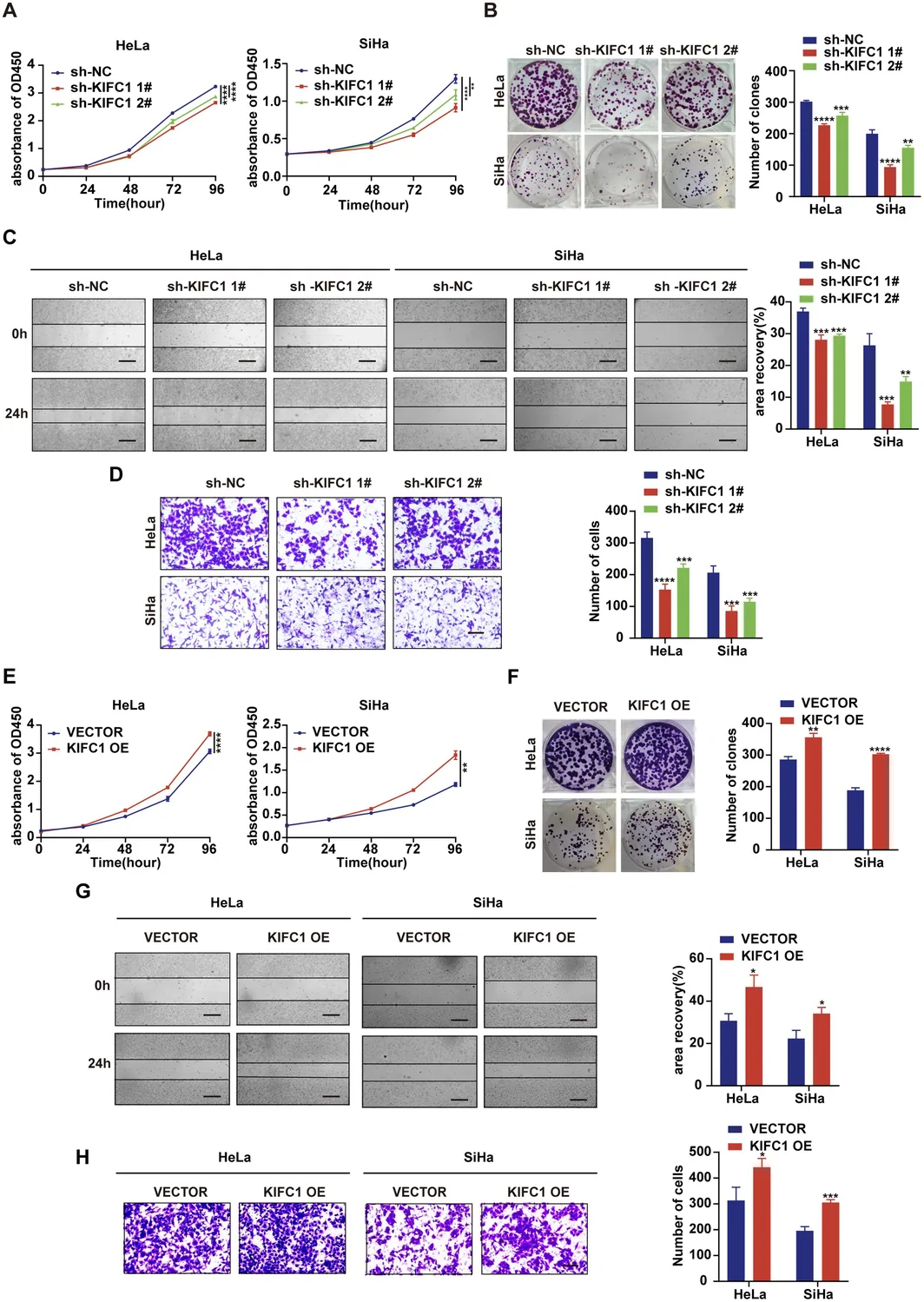
KIFC1 regulates the proliferation and migration of cervical cancer cells. (A, B) CCK‐8 and colony formation assays were performed to assess the proliferative capacity of sh‐KIFC1‐transfected cervical cancer cells (*n* = 3). (C, D) Wound healing and Transwell assays of cervical cancer cells following sh‐NC or sh‐KIFC1 transfection (scale bar: 100 μm). (E, F) Proliferative effects of KIFC1 overexpression in cervical cancer cells using CCK‐8 and colony formation assays (*n* = 3). (G, H) Migratory ability of KIFC1‐overexpressing cervical cancer cells evaluated using wound healing and Transwell assays (scale bar: 100 μm). Data are expressed as mean ± SD. Experiments were performed in triplicate. *p < 0.05, **p < 0.01, ***p < 0.001, ****p < 0.0001.

CCK‐8 and colony formation assays demonstrated that KIFC1 knockdown significantly inhibited cervical cancer cell proliferation (Figure 2A,B). We next assessed the effect of KIFC1 on cell motility. Wound healing and Transwell assays revealed that KIFC1 depletion reduced the migratory capacity of cervical cancer cells (Figure 2C,D). Conversely, KIFC1 overexpression enhanced cell proliferation, colony formation and migration in both HeLa and SiHa cells (Figure 2E–H). Collectively, these findings indicate that KIFC1 promotes malignant phenotypes in cervical cancer.

### 3.3 KIFC1 Regulates Glycolytic Metabolism and the Expression of PKM Isoforms in Cervical Cancer Cells

**FIGURE 3:**
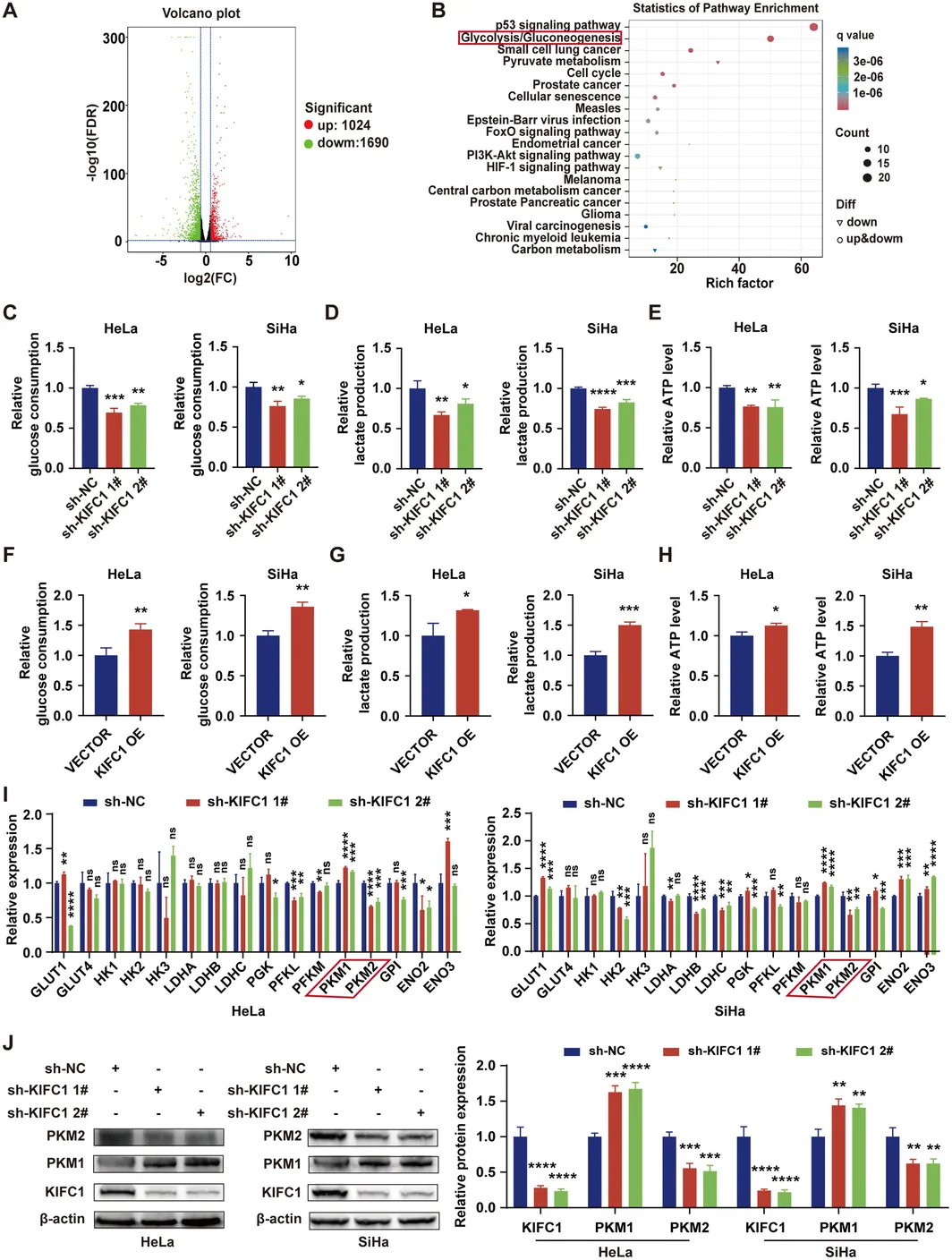
KIFC1 promotes cervical cancer glycolysis via PKM1/PKM2. (A) Volcano plot of genes altered by sh‐KIFC1 (*n* = 3). (B) KEGG analysis identified enriched pathways in cervical cancer cells after KIFC1 knockdown. (C–E) Quantification of glucose consumption, lactate production and ATP levels in sh‐KIFC1 cervical cancer cells (*n* = 3). (F–H) Glucose consumption, lactate production and ATP levels were measured in cervical cancer cells after KIFC1 overexpression (*n* = 3). (I) The expression of key glycolytic enzymes in cervical cancer cells upon KIFC1 knockdown by RT‐qPCR (*n* = 3). (J) Western blot analysis of PKM1 and PKM2 levels in KIFC1‐knockdown cells (*n* = 3). Data are expressed as mean ± SD. Experiments were performed in triplicate. *p < 0.05, **p < 0.01, ***p < 0.001, ****p < 0.0001.

To further investigate the mechanism by which KIFC1 regulates cervical cancer behaviour, we used RNA‐seq in HeLa cells following KIFC1 knockdown. A total of 2714 differentially expressed genes (DEGs) were identified (fold change ≥ 1.5, FDR < 0.05), including 1024 upregulated and 1690 downregulated genes (Figure 3A). KEGG pathway analysis revealed significant enrichment of metabolic pathways, including glycolysis and gluconeogenesis, suggesting that KIFC1 may be involved in metabolic regulation (Figure 3B). Although the p53 signalling pathway was also enriched, we focused on glycolysis due to its consistent enrichment and functional relevance. Consistent with this observation, glucose consumption, lactate production and ATP levels were significantly altered by KIFC1 modulation, indicating that KIFC1 is involved in glycolytic metabolism (Figure 3C–H).

Notably, RNA‐seq analysis showed a reduction in total PKM expression following KIFC1 knockdown. Given that bulk RNA‐seq does not distinguish between PKM1 and PKM2 isoforms, we further analysed representative glycolytic genes and PKM isoform expression by RT‐qPCR, including GLUT1, GLUT4, HK1, HK2, HK3, LDHA, LDHB, LDHC, PGK, PFKL, PFKM, PKM1, PKM2, GPI, ENO2 and ENO3. Interestingly, KIFC1 knockdown increased PKM1 expression while decreasing PKM2 expression, suggesting a shift in the balance of PKM isoforms. The overall reduction in total PKM expression is likely attributable to the predominant decrease in PKM2, the major isoform in cancer cells. Western blot analysis further confirmed these findings at the protein level in sh‐KIFC1 cells (Figure 3I,J). Collectively, these results suggest that KIFC1 may regulate glycolytic metabolism, at least in part, through modulating PKM isoform expression in cervical cancer cells.

### 3.4 KIFC1 Regulates SRSF3 Subcellular Localization in Cervical Cancer Cells

**FIGURE 4:**
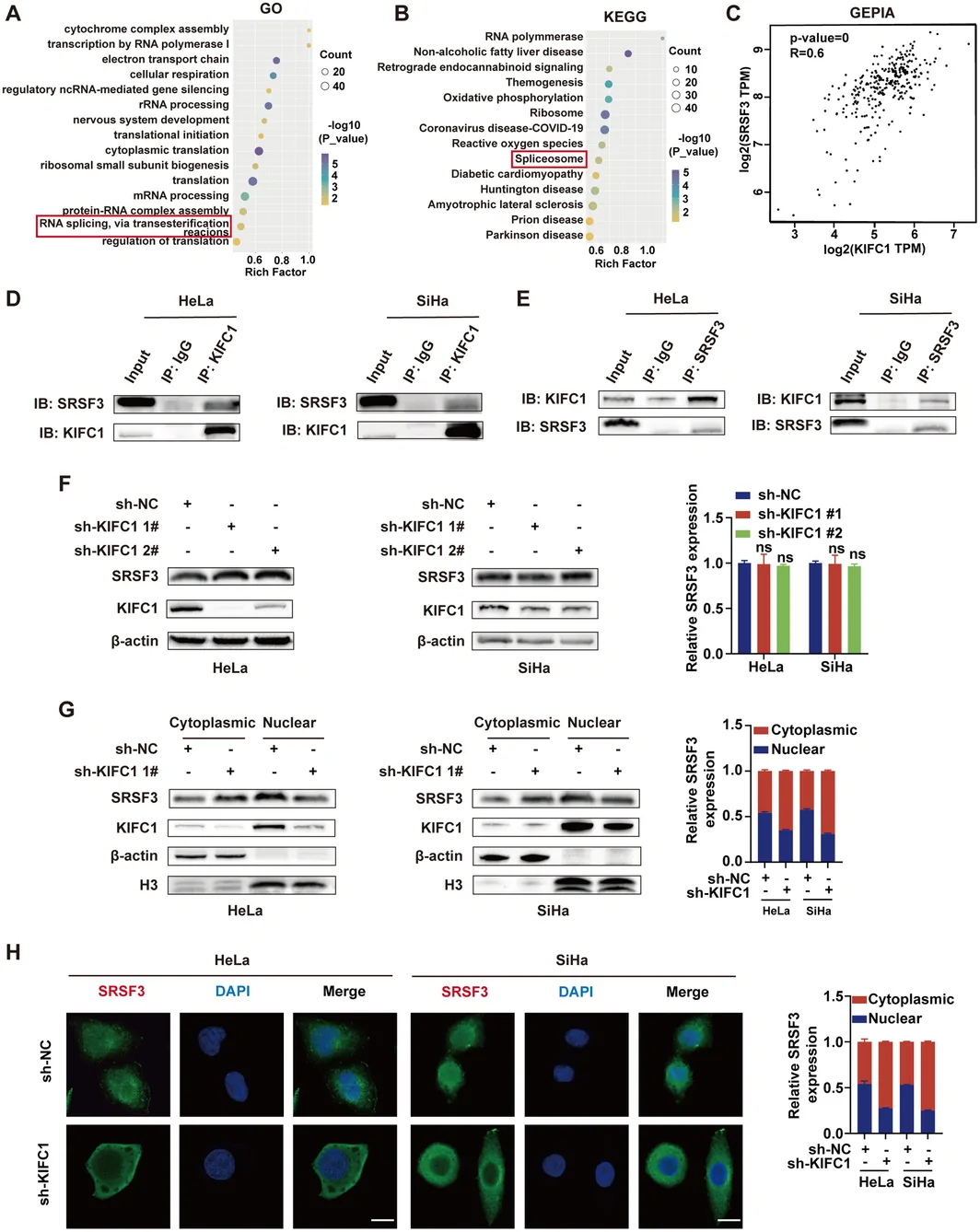
KIFC1 regulates the subcellular localization of SRSF3 in cervical cancer cells. (A, B) GO and KEGG pathway enrichment of KIFC1‐interacting proteins identified by Co‐IP/MS. (C) Correlation between KIFC1 and SRSF3 by the GEPIA database. (D, E) Co‐IP assay showing the interaction between KIFC1 and SRSF3 (*n* = 3). (F) Western blot analysis of SRSF3 levels in cervical cancer cells following KIFC1 silencing (*n* = 3). (G) Assessment of SRSF3 in the cytoplasm and nucleus upon KIFC1 knockdown using nuclear and cytoplasmic fractionation and Western blot (*n* = 3). (H) Confocal immunofluorescence microscopy showing altered subcellular localization of SRSF3 (scale bar: 20 μm). Data are expressed as mean ± SD. Experiments were performed in triplicate. *p < 0.05, **p < 0.01, ***p < 0.001, ****p < 0.0001.

We performed Co‐IP/MS and identified 1067 proteins interacting with KIFC1. KEGG and GO enrichment analyses revealed multiple significantly enriched pathways (Figure 4A,B). Notably, spliceosome components and mRNA splicing‐related processes were enriched, although they were not among the top‐ranked categories. Given our prior findings that KIFC1 regulates PKM isoform switching, we therefore prioritized these pathways for further investigation. Among the identified candidates, we focused on SRSF3, a splicing factor known to regulate mRNA splicing. GEPIA2 database analysis showed a robust correlation between KIFC1 and SRSF3 in cervical cancer, which was further supported by Co‐IP experiments demonstrating their interaction in cervical cancer cells (Figure 4C–E).

To determine whether KIFC1 regulates SRSF3 expression, RT‐qPCR and Western blot analysis showed that total SRSF3 expression was not significantly altered (Figure 4F and Figure S3A). We therefore investigated whether KIFC1 influences the subcellular distribution of SRSF3. Nuclear and cytoplasmic fractionation showed that KIFC1 knockdown reduced nuclear SRSF3 while increasing its cytoplasmic accumulation (Figure 4G). These results were further confirmed by confocal immunofluorescence microscopy (Figure 4H). Collectively, these findings indicate that KIFC1 interacts with SRSF3 and regulates its subcellular localization in cervical cancer cells.

### 3.5 SRSF3 May Regulate PKM Alternative Splicing and Contribute to Malignant Phenotypes in Cervical Cancer Cells

**FIGURE 5:**
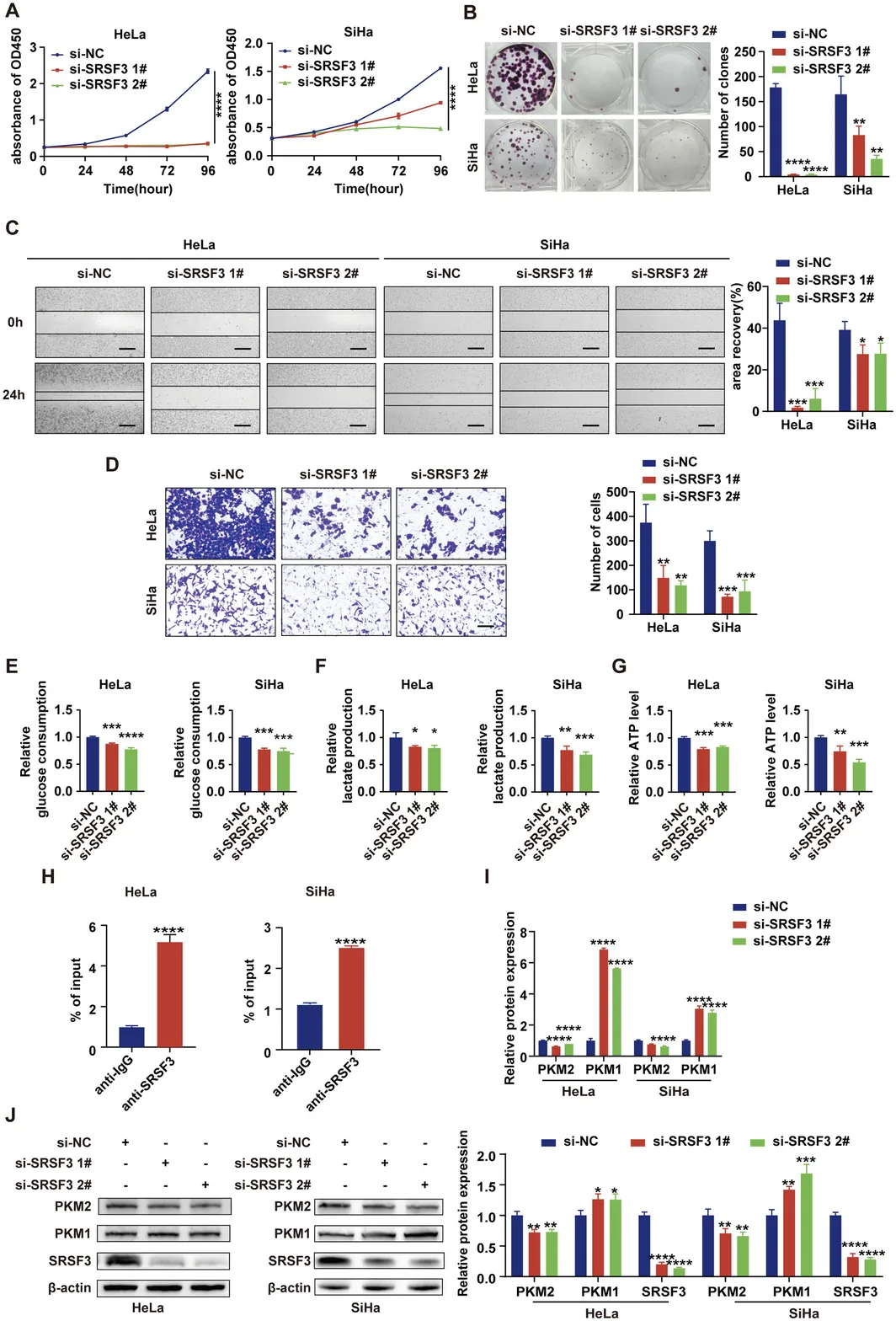
SRSF3 may regulate PKM alternative splicing and contribute to malignant phenotypes in cervical cancer cells. (A, B) CCK‐8 and colony formation assays demonstrating the impact of SRSF3 silencing on HeLa and SiHa cell proliferation (*n* = 3). (C, D) Migratory capacity following SRSF3 depletion through wound healing and Transwell assays (scale bar: 100 μm). (E–G) Glucose consumption, lactate production and ATP levels were measured in si‐SRSF3 cells (*n* = 3). (H) RIP assays showed that SRSF3 bound to PKM mRNA in cervical cancer cells (*n* = 3). (I) RT‐qPCR was performed to quantify PKM1 and PKM2 expression in SRSF3‐knockdown cells (*n* = 3). (J) Western blot analysis of PKM1 and PKM2 expression in si‐SRSF3 cells (*n* = 3). Data are expressed as mean ± SD. Experiments were performed in triplicate. *p < 0.05, **p < 0.01, ***p < 0.001, ****p < 0.0001.

To elucidate the role of SRSF3 in cervical cancer, HeLa and SiHa cells were subjected to transient SRSF3 silencing. RT‐qPCR analysis confirmed a significant reduction in SRSF3 expression in si‐SRSF3 cells, which was further validated by Western blot (Figure S4A,B). CCK‐8 and colony formation assays demonstrated reduced proliferation in SRSF3‐knockdown cell lines (Figure 5A,B). Wound healing and Transwell assays showed suppressed wound closure and cell migration in cell lines with SRSF3 knockdown (Figure 5C,D). Further analysis revealed that glucose consumption, lactate production, and ATP levels were significantly reduced by SRSF3 silencing (Figure 5E–G). Collectively, these findings indicate that SRSF3 is essential for sustaining the proliferative, migratory, and glycolytic capacity of cervical cancer cells.

Given the known role of SRSF3 in alternative splicing and the importance of PKM isoform in glycolytic regulation, we hypothesized that SRSF3 may regulate PKM alternative splicing in cervical cancer. RIP assays demonstrated strong binding of SRSF3 to PKM mRNA in cervical cancer cells (Figure 5H). Furthermore, RT‐qPCR and Western blot analysis revealed that SRSF3 knockdown resulted in increased PKM1 and decreased PKM2 levels in cervical cancer cells (Figure 5I,J). In summary, SRSF3 may regulate PKM alternative splicing, thereby contributing to glycolytic and malignant phenotypes in cervical cancer.

### 3.6 SRSF3 Restoration Partially Rescues the Malignant Phenotypes and Glycolytic Alterations Induced by KIFC1 Depletion

**FIGURE 6:**
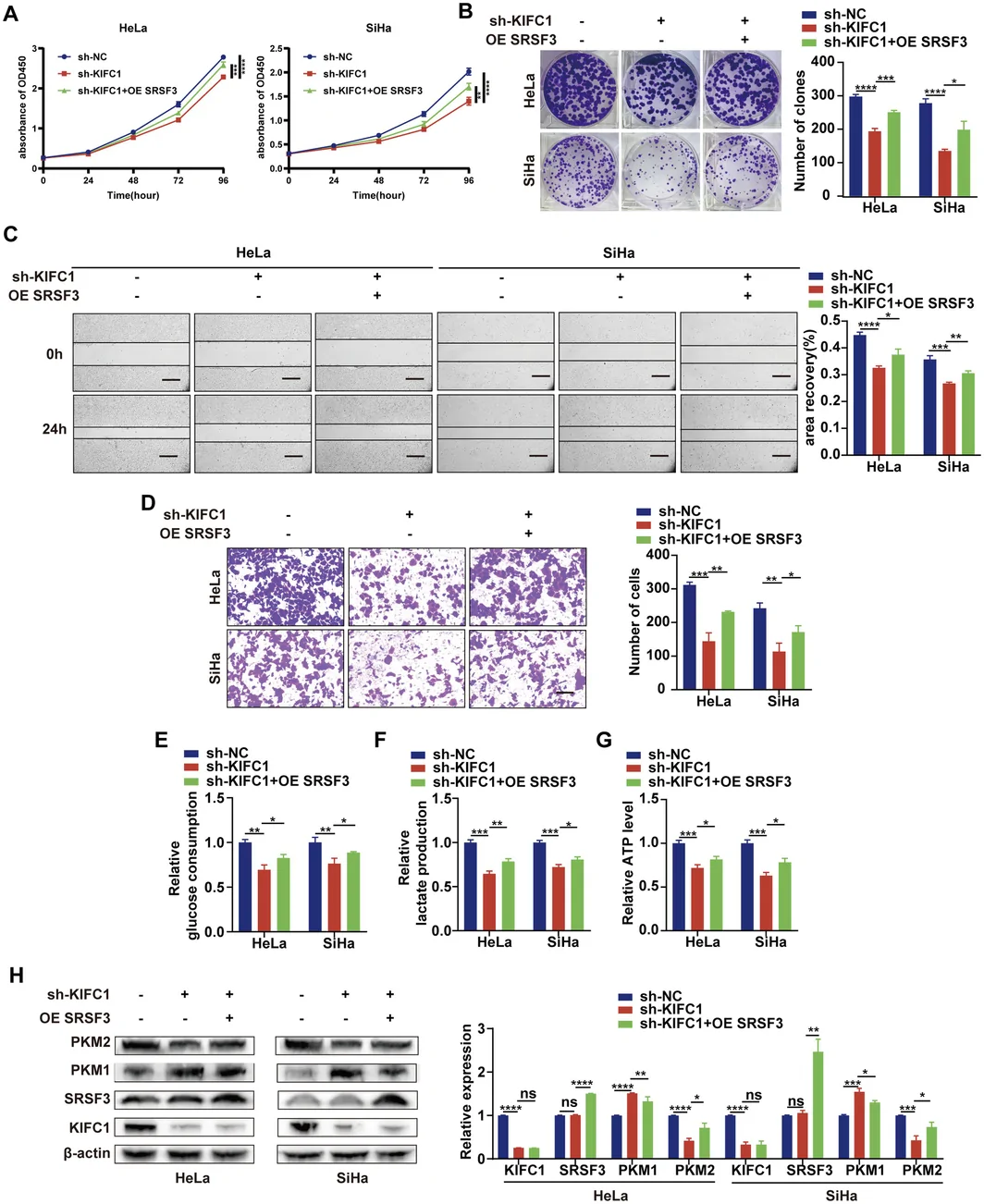
SRSF3 restoration partially rescues the malignant phenotypes and glycolytic alterations induced by KIFC1 depletion. (A, B) CCK‐8 and colony formation assays of HeLa and SiHa cells transduced with sh‐NC, sh‐KIFC1 or sh‐KIFC1 + OE SRSF3 (*n* = 3). (C, D) Wound healing and Transwell migration assays of cervical cancer cells in the indicated groups (*n* = 3). (E–G) Glucose consumption, lactate production and intracellular ATP levels in HeLa and SiHa cells in the indicated groups (*n* = 3). (H) Western blot analysis of KIFC1, SRSF3, PKM1 and PKM2 expression in the indicated groups (*n* = 3). Data are expressed as mean ± SD. Experiments were performed in triplicate. *p < 0.05, **p < 0.01, ***p < 0.001, ****p < 0.0001.

To further determine whether SRSF3 mediates the biological effects of KIFC1 in cervical cancer cells, we performed rescue experiments by restoring SRSF3 expression in KIFC1‐depleted HeLa and SiHa cells. Cells were assigned to three groups: sh‐NC, sh‐KIFC1 and sh‐KIFC1 + OE SRSF3. Western blot analysis confirmed efficient KIFC1 depletion and SRSF3 restoration in the corresponding groups (Figure 6H).

CCK‐8 and colony formation assays revealed a significant reduction in cell proliferation following KIFC1 depletion, which was partially reversed by SRSF3 restoration (Figure 6A,B). Consistent with these findings, wound healing and Transwell assays demonstrated that the impaired migratory capacity caused by KIFC1 depletion was partially restored upon SRSF3 overexpression (Figure 6C,D).

We next examined whether SRSF3 restoration could alleviate the glycolytic alterations associated with KIFC1 depletion. Glucose consumption, lactate production and intracellular ATP levels were all significantly decreased following KIFC1 knockdown, whereas SRSF3 restoration partially restored these metabolic parameters (Figure 6E–G). In addition, Western blot analysis revealed that KIFC1 depletion reduced PKM2 protein expression while increasing PKM1 protein expression; these changes were partially reversed by SRSF3 restoration (Figure 6H).

Taken together, these findings suggest that SRSF3 contributes to the biological effects of KIFC1 and that restoration of SRSF3 partially rescues the malignant and glycolytic phenotypes induced by KIFC1 depletion in cervical cancer cells.

### 3.7 KIFC1 Regulates Radiosensitivity of Cervical Cancer Cells Through the SRSF3/PKM2 Glycolytic Axis

**FIGURE 7:**
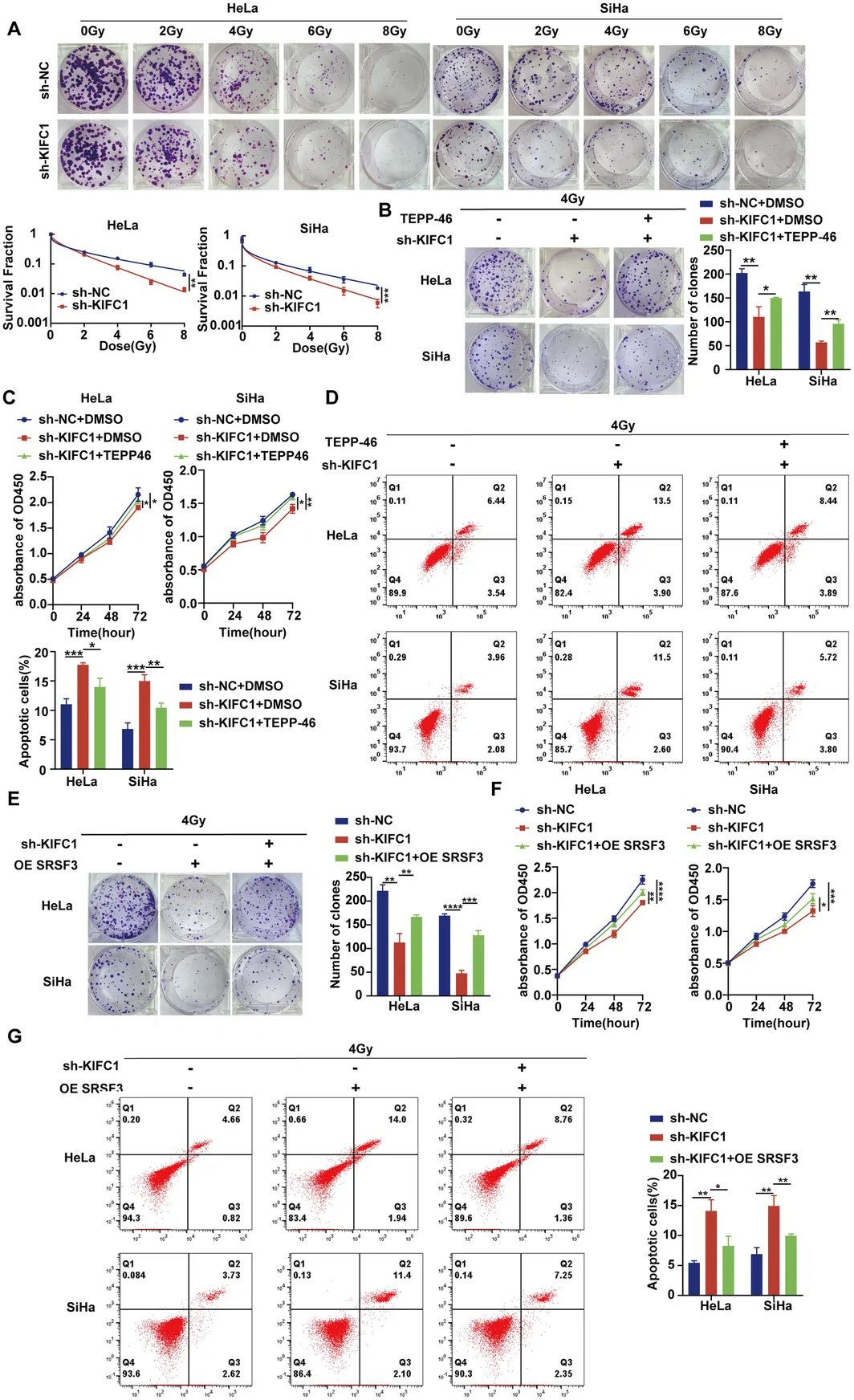
KIFC1 depletion enhances radiosensitivity of cervical cancer cells by PKM2‐dependent glycolysis. (A) Clonogenic survival curve of KIFC1‐silenced cervical cancer cells exposed to the indicated doses of irradiation (*n* = 3). (B, C) CCK‐8 assay and colony formation assay of HeLa and SiHa cells after KIFC1 knockdown, with or without TEPP‐46 treatment, under 4 Gy irradiation (*n* = 3). (D) Apoptosis in irradiated cervical cancer cells after KIFC1 knockdown, with or without TEPP‐46 treatment (*n* = 3). (E, F) Colony formation and CCK‐8 assays of HeLa and SiHa cells transduced with sh‐NC, sh‐KIFC1 or sh‐KIFC1 + OE SRSF3 under 4 Gy irradiation (*n* = 3). (G) Flow cytometric analysis of apoptosis in HeLa and SiHa cells transduced with sh‐NC, sh‐KIFC1 or sh‐KIFC1 + OE SRSF3 under 4 Gy irradiation (*n* = 3). Data are expressed as mean ± SD. Experiments were performed in triplicate. *p < 0.05, **p < 0.01, ***p < 0.001, ****p < 0.0001.

Given that the Warburg effect functions as a critical metabolic feature that drives radioresistance, targeting aerobic glycolysis represents a promising strategy to enhance radiosensitivity [11]. To investigate the role of KIFC1 in regulating cervical cancer cell radiosensitivity, HeLa and SiHa cells were exposed to graded doses of irradiation (0, 2, 4, 6 and 8 Gy), followed by colony formation assays to evaluate clonogenic survival. Compared with control cells, KIFC1‐depleted cells exhibited a significantly reduced survival fraction at multiple radiation doses, particularly at 4 Gy and above, indicating enhanced radiosensitivity (Figure 7A).

Based on the clonogenic survival curves, 4 Gy was selected for subsequent experiments, as it produced a clear and reproducible difference in survival between control and KIFC1‐depleted cells while maintaining sufficient colony formation for quantitative analysis. To determine whether this effect is mediated through PKM2‐dependent glycolysis, cervical cancer cells were exposed to TEPP‐46, a selective PKM2 activator. Under 4 Gy irradiation, KIFC1 knockdown markedly suppressed cell proliferation, as evidenced by colony formation and CCK‐8 assays, whereas TEPP‐46 treatment partially restored proliferative activity (Figure 7B,C). Moreover, flow cytometry analysis indicated that KIFC1 knockdown significantly enhanced radiation‐induced apoptosis, while TEPP‐46 treatment attenuated this effect to some extent (Figure 7D).

To further determine whether SRSF3 contributes to the regulation of radiosensitivity downstream of KIFC1, we performed rescue experiments by restoring SRSF3 expression in KIFC1‐depleted cervical cancer cells. Under 4 Gy irradiation, KIFC1 knockdown significantly reduced clonogenic survival and cell proliferation, whereas SRSF3 restoration partially reversed these effects, as demonstrated by colony formation and CCK‐8 assays (Figure 7E,F). Consistently, KIFC1 depletion markedly increased radiation‐induced apoptosis, while restoration of SRSF3 partially attenuated the apoptotic response (Figure 7G). These findings indicate that SRSF3 restoration partially rescues the radiosensitivity phenotype induced by KIFC1 depletion, supporting the involvement of the KIFC1/SRSF3 pathway in the regulation of cervical cancer radiosensitivity.

Taken together, these findings suggest that KIFC1 depletion enhances the radiosensitivity of cervical cancer cells, at least in part, through disruption of the SRSF3/PKM2‐mediated glycolytic pathway, whereas restoration of SRSF3 partially reverses the radiosensitizing effect of KIFC1 depletion.

### 3.8 KIFC1 Depletion Enhances Radiosensitivity and Radiation‐Induced DNA Damage Through the SRSF3/PKM2 Axis

**FIGURE 8:**
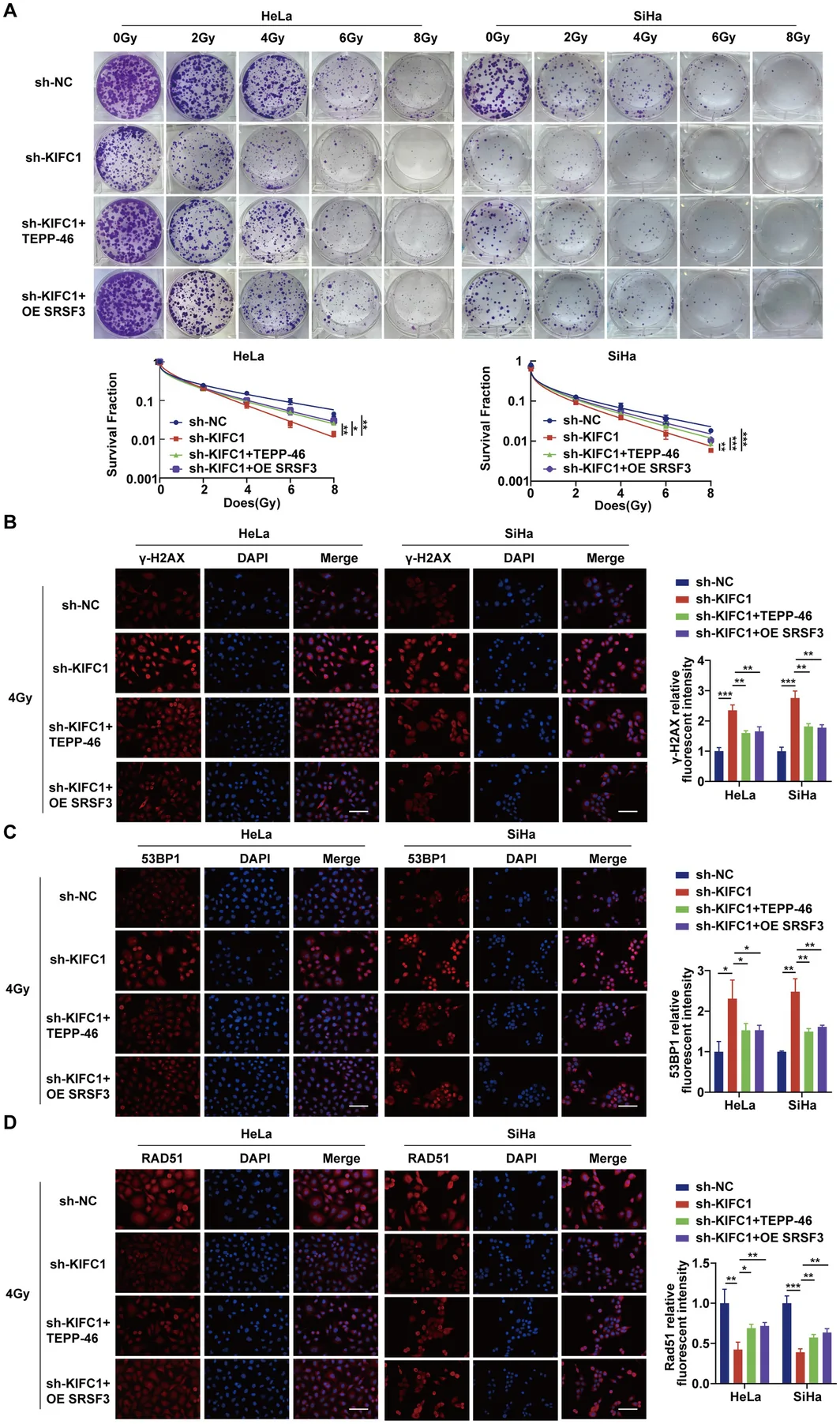
KIFC1 depletion enhances radiosensitivity and radiation‐induced DNA damage through the SRSF3/PKM2 axis. (A) Clonogenic survival curves of HeLa and SiHa cells transduced with sh‐NC, sh‐KIFC1, sh‐KIFC1 + OE SRSF3 or sh‐KIFC1 + TEPP‐46 and exposed to the indicated doses of irradiation (0, 2, 4, 6 and 8 Gy) (*n* = 3). (B) Representative immunofluorescence images and quantification of γ‐H2AX fluorescence intensity in HeLa and SiHa cells in the indicated groups after 4 Gy irradiation (scale bar: 50 μm). (C) Representative immunofluorescence images and quantification of 53BP1 fluorescence intensity in the indicated groups after 4 Gy irradiation (scale bar: 50 μm). (D) Representative immunofluorescence images and quantification of RAD51 fluorescence intensity in the indicated groups after 4 Gy irradiation (scale bar: 50 μm). Data are expressed as mean ± SD. Experiments were performed in triplicate. *p < 0.05, **p < 0.01, ***p < 0.001, ****p < 0.0001.

To further determine whether the SRSF3/PKM2 axis contributes to the radiosensitizing effect of KIFC1 depletion, we performed rescue experiments by restoring SRSF3 expression or activating PKM2. HeLa and SiHa cells were transduced with sh‐NC, sh‐KIFC1, sh‐KIFC1 + OE SRSF3 or sh‐KIFC1 + TEPP‐46 and exposed to graded doses of irradiation (0, 2, 4, 6 and 8 Gy), followed by clonogenic survival assays. Compared with sh‐NC cells, KIFC1‐depleted cells exhibited a markedly reduced surviving fraction across the indicated radiation doses, confirming enhanced radiosensitivity following KIFC1 depletion. Importantly, restoration of SRSF3 partially increased clonogenic survival in KIFC1‐depleted cells, whereas TEPP‐46 treatment similarly attenuated the radiosensitizing effect of KIFC1 depletion (Figure 8A). These findings further support the involvement of the SRSF3/PKM2 axis in KIFC1‐mediated regulation of radiosensitivity.

We next investigated whether KIFC1 depletion was associated with enhanced radiation‐induced DNA damage. Following 4 Gy irradiation, immunofluorescence analysis revealed a significant increase in γ‐H2AX fluorescence intensity in KIFC1‐depleted cells compared with sh‐NC cells, indicating enhanced radiation‐induced DNA damage. Notably, restoration of SRSF3 partially attenuated the increase in γ‐H2AX fluorescence intensity induced by KIFC1 depletion, while TEPP‐46 treatment also partially reduced γ‐H2AX signal intensity (Figure 8B).

We further examined 53BP1 and RAD51 to characterize the DNA damage response and DNA repair‐associated changes following irradiation. Compared with sh‐NC cells, KIFC1 depletion significantly increased 53BP1 fluorescence intensity while decreasing RAD51 fluorescence intensity after 4 Gy irradiation. These changes were partially reversed by either SRSF3 restoration or TEPP‐46 treatment (Figure 8C,D). Collectively, increased γ‐H2AX and 53BP1 fluorescence intensities together with reduced RAD51 fluorescence intensity in KIFC1‐depleted cells suggest enhanced radiation‐induced DNA damage and alterations in DNA damage response and repair‐associated signalling. Restoration of SRSF3 or activation of PKM2 partially reversed these changes, further supporting the involvement of the SRSF3/PKM2 axis in KIFC1‐mediated radiosensitivity.

### 3.9 KIFC1 Depletion Inhibits Xenograft Progression and Enhances Radiosensitivity In Vivo

**FIGURE 9:**
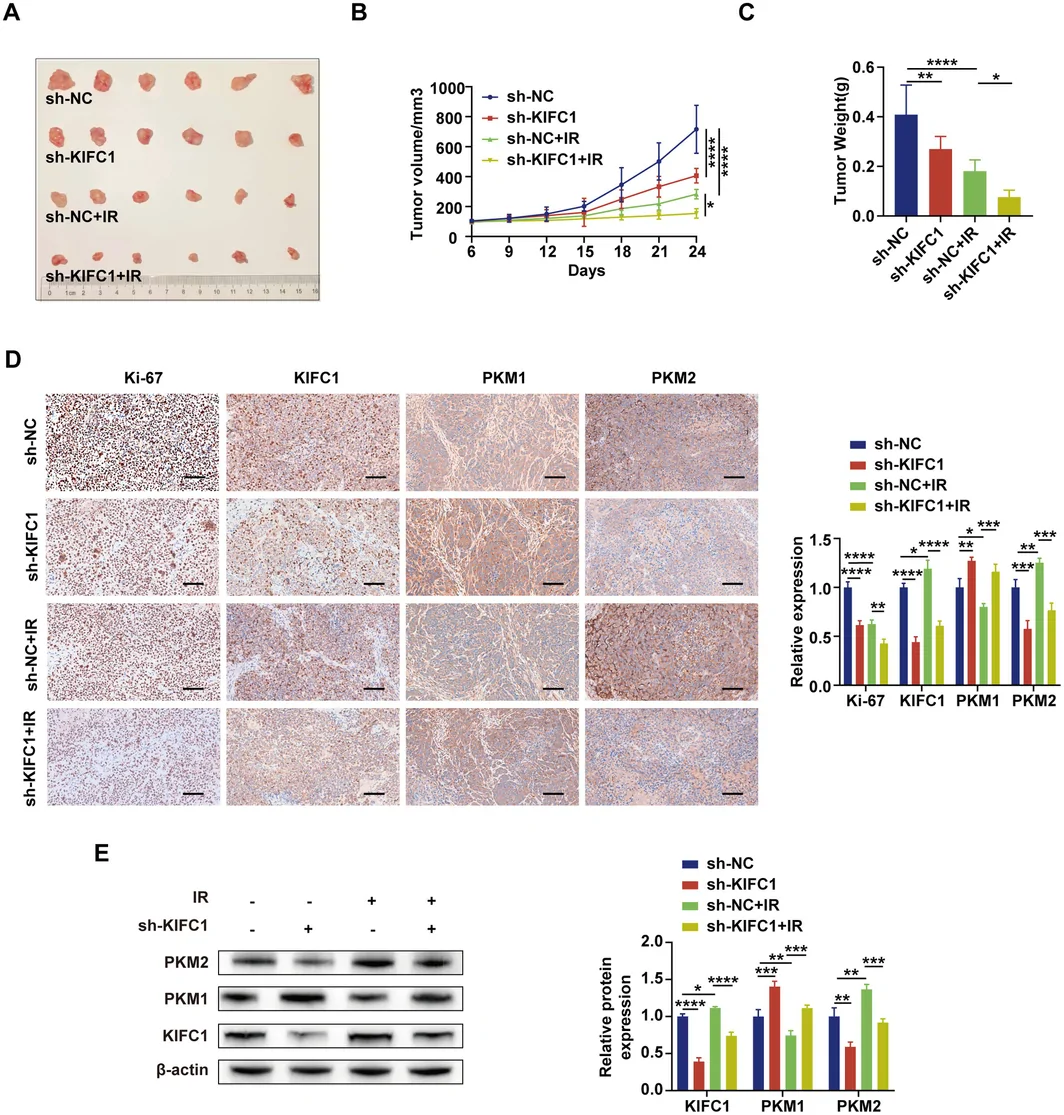
KIFC1 depletion inhibits xenograft progression and enhances radiosensitivity in vivo. (A) Representative images of tumour xenografts from KIFC1‐knockdown HeLa cells in nude mice after 6 Gy irradiation (*n* = 6). (B) Growth curves of xenograft tumours from control and KIFC1‐silenced HeLa cells in nude mice after 6 Gy irradiation (*n* = 6). (C) Tumour weights of xenografts derived from sh‐NC and sh‐KIFC1 HeLa cells after radiotherapy (*n* = 6). (D) Expression of Ki‐67, KIFC1, PKM1 and PKM2 in tumour tissues was evaluated by IHC analysis (scale bar: 100 μm; *n* = 6). (E) Protein levels of KIFC1, PKM1 and PKM2 in tumour tissues were assessed by Western blotting. Data are expressed as mean ± SD. Experiments were performed in triplicate. *p < 0.05, **p < 0.01, ***p < 0.001, ****p < 0.0001.

To further evaluate the role of KIFC1 in vivo, we subcutaneously xenografted KIFC1‐silenced and control cells into nude mice (Figure 9A and Figure S5A). Based on the in vitro findings, a radiation dose of 6 Gy was applied in vivo to ensure sufficient antitumor efficacy and measurable tumour growth inhibition. The tumour growth curves demonstrated that KIFC1 knockdown or radiotherapy as single treatments significantly suppressed tumour cell proliferation. Importantly, sh‐KIFC1 + IR resulted in significantly slower tumour growth compared with either treatment alone (Figure 9B). However, the overall magnitude of tumour growth inhibition in vivo was relatively moderate, suggesting a tumour growth delay effect rather than complete tumour regression. Consistently, tumour weight analysis further confirmed these findings (Figure 9C). Moreover, IHC analysis showed that KIFC1 knockdown, irradiation and their combination all resulted in decreased Ki‐67 expression in tumour xenografts, suggesting suppressed tumour cell proliferation and reduced malignancy. In addition, irradiation slightly increased KIFC1 and PKM2 expression while modestly decreasing PKM1 expression, whereas sh‐KIFC1 xenografts exhibited increased PKM1 and reduced PKM2. Similar expression patterns were also observed in tumours treated with the combination of sh‐KIFC1 and irradiation (Figure 9D). The results were validated via Western blot, which confirmed the changes in KIFC1, PKM1 and PKM2 (Figure 9E). These results suggest that KIFC1 may contribute to cervical cancer progression, potentially through modulation of PKM2‐associated glycolytic metabolism, and indicate its potential as a therapeutic target, particularly in combination with radiotherapy.

## 4 Discussion

Metabolic reprogramming towards aerobic glycolysis is increasingly recognized as a central determinant of tumour progression and therapeutic resistance [26, 27, 28]. In cervical cancer, accumulating evidence indicates that enhanced glycolytic flux supports malignant growth and confers protection against radiation‐induced damage. However, the upstream molecular mechanisms that integrate intracellular signalling, RNA processing and metabolic adaptation in response to radiotherapy have remained incompletely understood. Although KEGG analysis identified the p53 signalling pathway as one of the top enriched pathways, we focused on glycolysis‐related pathways for further investigation. This decision was based on the well‐established role of metabolic reprogramming in regulating radiosensitivity in cancer cells. Moreover, glycolysis‐associated pathways showed consistent enrichment and were strongly supported by our functional assays, including measurements of glucose consumption, lactate production and ATP levels. Therefore, glycolysis represents a more biologically relevant and experimentally supported mechanism underlying KIFC1 function. In this study, we identify KIFC1 as a previously unrecognized regulator that links intracellular transport to alternative splicing‐dependent metabolic reprogramming, thereby modulating radiosensitivity in cervical cancer.

Our data establish KIFC1 as an oncogenic factor that promotes cervical cancer progression through sustained glycolytic activity. Consistent with its overexpression in multiple malignancies, KIFC1 was markedly upregulated in cervical cancer tissues. Functional assays demonstrated that KIFC1 depletion suppresses tumour cell proliferation and migration both in vitro and in vivo, accompanied by a pronounced reduction in glucose consumption, lactate production and ATP generation. These findings indicate that KIFC1 is an important regulator of glycolytic phenotypes that may support cervical cancer growth.

A central mechanistic insight of this study is the identification of a KIFC1‐SRSF3‐PKM regulatory axis that links intracellular transport to alternative splicing‐driven metabolic control. PKM alternative splicing represents a key metabolic switch between oxidative metabolism (PKM1) and aerobic glycolysis (PKM2). While SRSF3 has been reported to promote PKM exon 10 inclusion and PKM2 expression, the upstream mechanisms governing its splicing activity in cancer cells have remained unclear [29, 30]. Our findings demonstrate that KIFC1 does not alter SRSF3 expression levels but instead governs its subcellular localization. By regulating the subcellular localization of SRSF3, KIFC1 shifts PKM isoform expression, thereby maintaining enhanced glycolytic activity in cervical cancer cells. This reveals an additional regulatory layer in which motor protein–mediated transport may influence splicing factor activity and metabolic adaptation.

Notably, our proteomic and enrichment analyses support this mechanistic framework by highlighting spliceosome‐ and RNA‐processing‐related pathways among KIFC1‐interacting proteins. The functional dependency of PKM splicing on SRSF3 nuclear localization was further confirmed by RNA immunoprecipitation and rescue experiments, underscoring the specificity of this axis. Together, these results support a previously unrecognized splicing‐metabolic axis, in which intracellular transport machinery influences metabolic regulation through alternative splicing control.

Beyond its role in tumour growth, this axis has direct implications for radiotherapy response. Glycolytic metabolism has been shown to enhance radioresistance by supporting DNA repair, maintaining redox balance and mitigating radiation‐induced oxidative stress [31, 32]. In this context, we demonstrate that KIFC1 depletion significantly increases radiosensitivity in cervical cancer cells, as evidenced by reduced clonogenic survival and increased apoptosis following irradiation. Importantly, pharmacological activation of PKM2 with TEPP‐46 partially rescues the radiosensitization induced by KIFC1 knockdown, indicating that KIFC1‐mediated regulation of radiosensitivity is at least partly dependent on PKM2‐driven glycolysis. These findings position metabolic reprogramming as a functional downstream effector of KIFC1 in the radiation response.

Our in vivo data further corroborate this model, showing that KIFC1 knockdown suppresses tumour growth and enhances the therapeutic efficacy of radiotherapy in xenograft models, accompanied by a shift in the balance of PKM isoforms. Together, these results suggest that KIFC1 may facilitate metabolic adaptation to radiation stress by maintaining a pro‐glycolytic splicing programme, thereby conferring a survival advantage.

Notably, although KIFC1 depletion markedly enhanced radiation‐induced apoptosis in vitro, the magnitude of tumour growth inhibition observed in vivo was relatively moderate. However, the combination of KIFC1 knockdown and radiotherapy still resulted in the greatest delay in tumour growth, indicating a tumour growth delay effect rather than complete tumour regression. This discrepancy between in vitro and in vivo findings may be attributed to the complexity of the tumour microenvironment, including hypoxia, stromal interactions and metabolic heterogeneity, which can attenuate treatment responses. In addition, metabolic plasticity in vivo may enable tumour cells to compensate for glycolytic inhibition by activating alternative energy pathways.

This study expands the functional repertoire of KIFC1 beyond its established role in centrosome clustering and mitotic regulation [18, 19]. While previous work has implicated KIFC1 in glycolytic regulation through transcriptional pathways in other cancers, our findings reveal a distinct mechanism whereby KIFC1 may control metabolism at the post‐transcriptional level via alternative splicing [33]. This motor protein–splicing–metabolism axis provides a conceptual framework that integrates intracellular transport with metabolic plasticity and therapeutic resistance.

Several limitations of this study should be acknowledged. Although our findings are supported by cell‐based and animal models, validation in larger clinical cohorts will be necessary to fully establish the prognostic and therapeutic relevance of KIFC1 in cervical cancer. In addition, while our data identify SRSF3 as a key downstream effector, the broader splicing network regulated by KIFC1 and its potential impact on other metabolic or stress‐response pathways warrant further investigation. Importantly, although SRSF3 has been reported to regulate PKM alternative splicing, the specific splicing sites mediating this process in cervical cancer were not directly examined in the present study and require further investigation. Finally, the precise molecular determinants governing the interaction between KIFC1 and SRSF3, including potential adaptor proteins or regulatory signals, remain to be elucidated.

## 5 Conclusions

**FIGURE 10:**
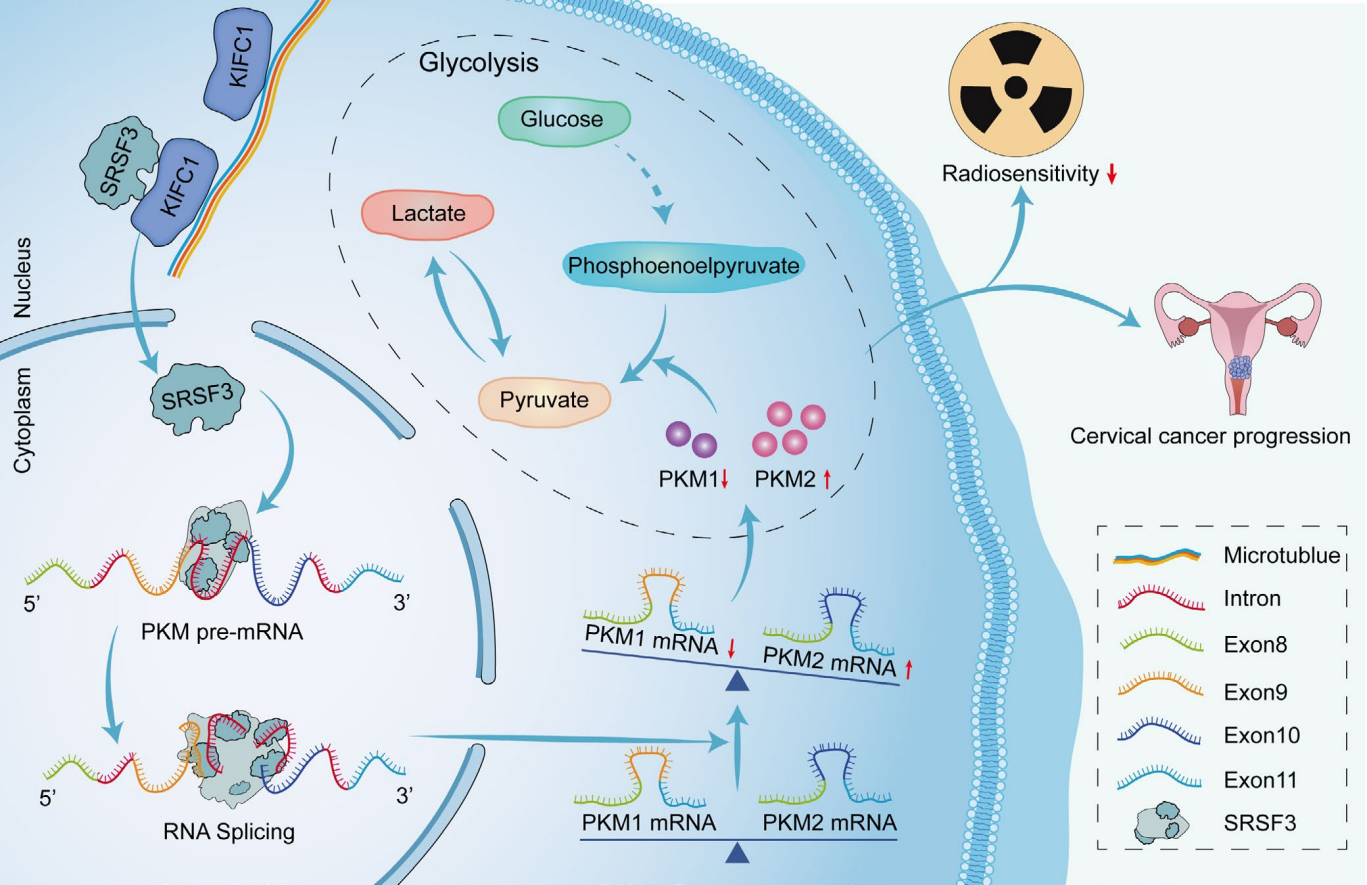
Schematic illustrating the function of KIFC1 in cervical cancer. KIFC1 regulates glycolysis by mediating the nuclear import of SRSF3 to modulate PKM alternative splicing, thereby promoting cervical cancer progression and reducing radiosensitivity.

In conclusion, our study suggests that KIFC1 may play an important role in cervical cancer progression and radiosensitivity, potentially involving a KIFC1‐SRSF3‐PKM regulatory axis (Figure 10). Our findings raise the possibility that KIFC1 may link intracellular transport to alternative splicing‐associated regulation of PKM isoforms and glycolytic reprogramming, thereby contributing to tumour growth and radioresistance. Targeting this pathway may represent a potential therapeutic strategy to disrupt metabolic adaptation and improve radiotherapy response in cervical cancer.

## Author Contributions

**Junxia Liu:** data curation, investigation, validation, writing – original draft. **Jiahao Li:** investigation, validation. **Haiting Zhou:** investigation, validation. **Wentao Ha:** validation, data curation. **Xinyu Wu:** validation, data curation. **Xiangdong Liu:** validation, data curation. **Yizhi Jiang:** validation, data curation. **Chen Gong:** writing – original draft. **Yi Cheng:** writing – original draft. **Qingxu Liu:** writing – original draft. **Tengfei Chao:** methodology, writing – review and editing, supervision, funding acquisition. **Huihua Xiong:** methodology, project administration, supervision, writing – review and editing.

## Funding

This work was supported by Natural Science Foundation of Hubei Province (Grant No. 2025AFD774).

## Ethics Statement

All animal experiments were approved by the Institutional Animal Ethics Committee of Tongji Hospital, Tongji Medical College, Huazhong University of Science and Technology (approval number: TJH‐202411043) and were conducted in strict accordance with the National Institutes of Health Guide for the Care and Use of Laboratory Animals. The use of human tissue samples was ethically reviewed and approved by the Ethics Committee of Shanghai Outdo Biotech Company (approval number: SHYJS‐CP‐1710003).

## Consent

The authors have nothing to report.

## Conflicts of Interest

The authors declare no conflicts of interest.

## Supporting information


**Figure S1:** Pan‐cancer expression landscape of KIFC1 and its association with cervical cancer subtypes and lymph node metastasis. (A) Pan‐cancer expression of KIFC1 across multiple tumour types using the TIMER 2.0 database. (B) KIFC1 expression in cervical cancer based on GEPIA2 database analysis. (C, D) UALCAN analysis of KIFC1 expression in cervical cancer subtypes and its correlation with lymph node metastasis. (E) KIFC1 in high‐ and low‐expression groups by IHC (scale bar: 50 μm).

**Figure S2:** Validation of KIFC1 knockdown and overexpression efficiency in cervical cancer cells. (A, B) KIFC1 knockdown efficiency in HeLa and SiHa cells assessed via RT‐qPCR and Western blot (*n* = 3). (C, D) RT‐qPCR and Western blot confirmation of KIFC1 overexpression efficiency in cervical cancer cells. *p < 0.05, **p < 0.01, ***p < 0.001, ****p < 0.0001.

**Figure S3:** Effect of KIFC1 knockdown on SRSF3 expression in cervical cancer cells. (A) RT‐qPCR quantitative analysis of SRSF3 levels in cervical cancer cells upon KIFC1 knockdown (*n* = 3). Data are expressed as mean ± standard deviation. Experiments were performed in triplicate. *p < 0.05, **p < 0.01, ***p < 0.001, ****p < 0.0001.

**Figure S4:** Validation of SRSF3 knockdown efficiency in cervical cancer cells. (A, B) SRSF3 knockdown efficiency in HeLa and SiHa cells assessed via RT‐qPCR and Western blot (*n* = 3). Data are expressed as mean ± standard deviation. Experiments were performed in triplicate. *p < 0.05, **p < 0.01, ***p < 0.001, ****p < 0.0001.

**Figure S5:** Representative images of tumour‐bearing mice from xenograft models. (A) Representative whole‐body images of nude mice bearing subcutaneous xenograft tumours established with cervical cancer cells in the following groups: sh‐NC, sh‐KIFC1, sh‐NC + IR and sh‐KIFC1 + IR. Images were captured at the experimental endpoint prior to tumour excision (*n* = 6).

**Table S1:** Primer sequences for mRNA detection.

## Data Availability

The data that support the findings of this study are available from the corresponding author upon reasonable request.
