# Supplementary material for: Targeting the KIFC1‐SRSF3‐PKM Axis Suppresses Cervical Cancer Glycolysis and Enhances Radiosensitivity
Source: J Cell Mol Med. 2026 Sep 20;30(18):e71373. doi: 10.1111/jcmm.71373 (PMC13590926; doi:10.1111/jcmm.71373)
Supplement: Supplementary file 1 [file JCMM-30-e71373-s002.docx]

**Supplementary materials**

**Targeting the KIFC1–SRSF3–PKM Axis Suppresses Cervical Cancer Glycolysis and Enhances Radiosensitivity**

**Authors:**

Junxia Liu^1^, Jiahao Li^1^, Haiting Zhou^1#^, Xinyu Wu^1^, Tengfei Chao^1*^ , Huihua Xiong^1*^

^1^Department of Oncology, Tongji Hospital, Tongji Medical College, Huazhong University of Science and Technology, Wuhan, Hubei, China.

^#^Department of Oncology, the First Affiliated Hospital of Nanchang University, Nanchang University, Nanchang, Jiangxi, China.

**^*^** Corresponding authors:

Tengfei Chao, No. 1095 Jiefang Avenue, Qiaokou District, Wuhan, Hubei Province, 430030, China, E-mail: [turnface@126.com](mailto:turnface@126.com), Tel: +86-27-8366 2688.

Huihua Xiong, No. 1095 Jiefang Avenue, Qiaokou District, Wuhan, Hubei Province, 430030, China, E-mail: [lizaabear@tjh.tjmu.edu.cn](mailto:lizaabear@tjh.tjmu.edu.cn), Tel: +86-27-8366 2688.

**Fig. S1. Pan-cancer expression landscape of KIFC1 and its association with cervical cancer subtypes and lymph node metastasis.**

**A.** Pan-cancer expression of KIFC1 across multiple tumor types using the TIMER 2.0 database. **B.** KIFC1 expression in cervical cancer based on GEPIA2 database analysis. **C-D.** UALCAN analysis of KIFC1 expression in cervical cancer subtypes and its correlation with lymph node metastasis. **E.** KIFC1 in high- and low-expression groups by IHC (scale bar: 50 μm).

**Fig. S2. Validation of KIFC1 knockdown and overexpression efficiency in cervical cancer cells.**

**A-B.** KIFC1 knockdown efficiency in HeLa and SiHa cells assessed via RT-qPCR and Western blot (n=3). **C-D.** RT-qPCR and Western blot confirmation of KIFC1 overexpression efficiency in cervical cancer cells. **p* < 0.05, ***p* < 0.01, ****p* < 0.001, *****p* < 0.0001.

**Fig. S3. Effect of KIFC1 knockdown on SRSF3 expression in cervical cancer cells.**

**A.** RT-qPCR quantitative analysis of SRSF3 levels in cervical cancer cells upon KIFC1 knockdown (n = 3). Data are expressed as mean ± standard deviation. Experiments were performed in triplicate. **p* < 0.05, ***p* < 0.01, ****p* < 0.001, *****p* < 0.0001.

**Fig. S4. Validation of SRSF3 knockdown efficiency in cervical cancer cells.**

**A-B.** SRSF3 knockdown efficiency in HeLa and SiHa cells assessed via RT-qPCR and Western blot (n=3). Data are expressed as mean ± standard deviation. Experiments were performed in triplicate. **p* < 0.05, ***p* < 0.01, ****p* < 0.001, *****p* < 0.0001.

**Fig. S5. Representative images of tumor-bearing mice from xenograft models.**

**A.** Representative whole-body images of nude mice bearing subcutaneous xenograft tumors established with cervical cancer cells in the following groups: sh-NC, sh-KIFC1, sh-NC + IR, and sh-KIFC1 + IR. Images were captured at the experimental endpoint prior to tumor excision (n=6).

**Table S1 Primer sequences for mRNA detection**

| Gene name | Forward（5^，^-3^，^） | Reverse（5^，^-3^，^） |
| --- | --- | --- |
| *GAPDH* | GGAGCGAGATCCCTCCAAAAT | GGCTGTTGTCATACTTCTCATGG |
| *KIFC1* | GGTGCAACGACCAAAATTACC | GGGTCCTGTCTTCTTGGAAAC |
| *SRSF3* | AATTGGAACGGGCTTTTGGC | CCATCTAGCTCTCGGACTGC |
| *GLUT1* | ATTGGCTCCGGTATCGTCAAC | GCTCAGATAGGACATCCAGGGTA |
| *GLUT4* | CCATCCTGATGACTGTGGCTCTC | GCCACGATGAACCAAGGAATGG |
| *HK1* | CTGCTGGTGAAAATCCGTAGTGG | GTCCAAGAAGTCAGAGATGCAGG |
| *HK2* | TGCCACCAGACTAAACTAGACG | CCCGTGCCCACAATGAGAC |
| *HK3* | CATCGTGGACTTCCAGCAGAAG | CTTGGTCCAGTTCAGGAGGATG |
| *LDHA* | ATGGCAACTCTAAAGGATCAGC | CCAACCCCAACAACTGTAATCT |
| *LDHB* | GGACAAGTTGGTATGGCGTGTG | AAGCTCCCATGCTGCAGATCCA |
| *LDHC* | AGAACATGGTGATTCTAGTGTGC | ACAGTCCAATAGCCCAAGAGG |
| *PGK* | TGGACGTTAAAGGGAAGCGG | GCTCATAAGGACTACCGACTTGG |
| *PFKL* | AAGAAGTAGGCTGGCACGACGT | GCGGATGTTCTCCACAATGGAC |
| *PFKM* | GCTTCTAGCTCATGTCAGACCC | CCAATCCTCACAGTGGAGCGAA |
| *PKM1* | GGAGAAACAGCCAAAGGGGACTA | CTGCCAGACTTGGTGAGGACG |
| *PKM2* | GGAGAAACAGCCAAAGGGGACTA | CTGCCAGACTCCGTCAGAACTATC |
| *GPI* | CTGGTAGACGGCAAGGATGTGA | TCCGTGATGGTCTTGCCTGTGT |
| *ENO2* | CTGTATCGCCACATTGCTCAGC | AGCTTGTTGCCAGCATGAGAGC |
| *ENO3* | CTGGTAGACGGCAAGGATGTGA | TCCGTGATGGTCTTGCCTGTGT |
